# Allergen homologs in the *Euroglyphus maynei* draft genome

**DOI:** 10.1371/journal.pone.0183535

**Published:** 2017-08-22

**Authors:** S. Dean Rider, Marjorie S. Morgan, Larry G. Arlian

**Affiliations:** Department of Biological Sciences, Wright State University, Dayton, Ohio, United States of America; University of Cincinnati, UNITED STATES

## Abstract

*Euroglyphus maynei* is a house dust mite commonly found in homes worldwide and is the source of allergens that sensitize and induce allergic reactions in humans. It is the source of species-specific allergens as well as allergens that are cross-reactive with the allergens from house dust mites *Dermatophagoides farinae* and *D*. *pteronyssinus*, and the ectoparasitic scabies mite *Sarcoptes scabiei*. The genomics, proteomics and molecular biology of *E*. *maynei* and its allergens have not been as extensively investigated as those of *D*. *farinae*, *D*. *pteronyssinus*, and *S*. *scabiei* where natural and recombinant allergens from these species have been characterized. Until now, little was known about the genome of *E*. *maynei* and it allergens but this information will be important for producing recombinant allergens for diagnostic and therapeutic purposes and for understanding the allergic response mechanism by immune effector cells that mediate the allergic reaction. We sequenced and assembled the 59 Mb *E*. *maynei* genome to aid the identification of homologs for known allergenic proteins. The predicted proteome shared orthologs with *D*. *farinae* and *S*. *scabiei*, and included proteins with homology to more than 30 different groups of allergens. However, the majority of allergen candidates could not be assigned as clear orthologs to known mite allergens. The genomic sequence data, predicted proteome, and allergen homologs identified from *E*. *maynei* provide insight into the relationships among astigmatid mites and their allergens, which should allow for the development of improved diagnostics and immunotherapy.

## Introduction

The allergy-causing house dust mite, *Euroglyphus maynei* occurs in homes worldwide [1–4]. It appears to be more common in homes in Europe and in the United Kingdom than in homes in the United States. Generally, *E*. *maynei* is less frequently found in homes than *Dermatophagoides farinae* and *D*. *pteronyssinus*. However, it usually co-inhabits homes with *D*. *farinae* and *D*. *pteronyssinus* and sometimes it is the most numerous of the three species or the only species found [5].

*E*. *maynei* is phylogenetically closely related to the common house dust mites, *D*. *farinae* and *D*. *pteronyssinus* and to the itch mite, *Sarcoptes scabiei*. *E*. *maynei* is an astigmatid mite and belongs to the suborder Oribata, infraorder Desmonomata and family Pyroglyphidae along with the *Dermatophagoides* spp [6,7].

Many patients that are sensitive to *D*. *farinae* and *D*. *pteronyssinus* are also sensitive to *E*. *maynei* [8–10]. *E*. *maynei* is the source of many allergens, some of which cross-react with those from *D*. *farinae* and *D*. *pteronyssinus* while others are unique to this mite species [9,11–15]. Antigens from *E*. *maynei* are also cross-reactive with some antigens from the scabies mite *S*. *scabiei*. However, there has been less research into *E*. *maynei* biology, allergens, prevalence of sensitivity and molecular biology of the allergens compared to *D*. *farinae* and *D*. *pteronyssinus*. Presumably, this is because this species is not as prevalent in homes as *D*. *farinae* and *D*. *pteronyssinus* and it is not widely cultured for research and commercial purposes. Thus, few of its allergens have been characterized or produced by recombinant technology. A draft genome for *D*. *farinae* has been produced [16] but none have been reported for *D*. *pteronyssinus* and *E*. *maynei*. The genomes have been sequenced for the related itch mite, *S*. *scabiei*, the unrelated mites, *Tetranychus urticae* and *Metaseiulus occidentalis* and the tick, *Ixodes scapularis* [7,17–19]. We report here the draft genome of *E*. *maynei* and compared its allergome to those of *D*. *farinae* and *D*. *pteronyssinus* and to the genomes of these other acarine species.

## Materials and methods

The strain of *E*. *maynei* that was chosen for genome sequencing is a long-standing laboratory reference culture that originated in Galveston, TX, USA, and has been maintained as a randomly-breeding population for over 20 years [5,20]. Genomic DNA was isolated from a pool of living mites (~20 mg wet weight) using the Wizard SV genomic DNA purification system (Promega, Madison, WI) and the manufacturer’s animal tissue protocol. Mites were ground in ice-cold digestion buffer using a Dounce homogenizer prior to an overnight proteinase K digestion and column purification of the DNA. TruSeq Library construction and paired end sequencing were done by Beckman Coulter Genomics (Danvers, MA) to generate ~24 million paired reads (2x150 bp; ~12 million fragments; 350 bp insert size). This was designed to cover ~100 Mb of DNA at over 30x coverage.

Unless specifically indicated, the software programs were used with the recommended or default settings. KmerGenie v1.6741 [21] was used to estimate the best value of k and the minimum abundance (m) for a given Kmer when using De Bruijn graph assembly algorithms. The best estimates provided were k = 47 and m = 7 using the diploid model or k = 53 and m = 12 with the regular model, although there were other peaks with the regular model. Therefore, different values of k (21, 47, 53, 71, 91) were used with their corresponding minimum abundance estimates to generate multiple assemblies. Minia v1.6088; [22] was used with SSPACE-STANDARD v3.0 [23] to assemble trimmed reads into gapped contigs. Within SSPACE, Bowtie v0.12.5 [24] was used to map reads during “scaffolding” and without implementing contig extension. The insert size provided to SSPACE was 372 +/- 334.8 bp. An additional assembly was also created using CLC Genomics Workbench v7.5. This method adopted a kmer size of 25, which was automatically implemented by the software to optimize computational efficiency based on the amount of input data.

The MISA perl script [25] was used to scan the assembly for simple sequence repeats of: 1 nt with >10 copies, 2 nt with >6 copies, 3 nt with >5 copies, 4 nt with >5 copies, 5 nt with >5 copies, 6 nt with >5 copies, 7 nt with >5 copies, and 8 nt with >5 copies.

The Benchmarking Universal Single-Copy Orthologs (BUSCO) strategy was used to test the completeness of the assemblies using the arthropod, metazoan, and eukaryote profiles [26]. The Augustus gene model output from the BUSCO arthropod analysis (in optimization mode) was used as input for gene predictions within the Maker software pipeline.

Maker software was implemented with the accessory software programs RepeatMasker v1.317 [27], SNAP [28], and Augustus v3.0.1 [29]. SNAP utilized a scabies mite Hidden Markov Model (HMM) generated previously [7] and Augustus used the BUSCO-generated model from the k = 47, m = 7 Minia assembly. Hints for gene predictions were based on 17,091 EST sequences from other Sarcoptiformes mites (NCBI EST database), and the CEGMA 458 core proteins [30]. Over 200 of the Maker predictions possessed introns less than 10 bp. These protein predictions were removed from the final annotation data set that was deposited into NCBI under BioProject PRJNA350546. Annotations of the mitochondrial genome contig were aided by the MITOS web server (http://mitos.bioinf.uni-leipzig.de/index.py) and RNA Weasel (http://megasun.bch.umontreal.ca/RNAweasel/). GAG software (http://genomeannotation.github.io/GAG) was used to analyze the output from Maker to quantify the number of loci with start and stop codons, and to generate tables needed for submission of the information into public databases.

Reciprocal best BLAST hits were identified using Legacy BLAST and the perl script orthoparahomlist.pl [31] and output was used to estimate the number of proteins that are shared among *E*. *maynei*, *S*. *scabiei* and *D*. *farinae*. Blast2Go was used to categorize proteins into functional groups based on the presence of conserved domains [32].

To identify candidate allergen proteins, allergen protein sequences from *Dermatophagoides farinae*, *D*. *pteronyssinus*, *Sarcoptes scabiei*, *Blomia tropicalis*, and *Euroglyphus maynei* were used as queries in BLAST searches against the predicted proteome of *E*. *maynei*. Query cutoffs included an expect of (E = 1x10^-5^) and a query coverage of >50%. Some fragments were later selected with lower query coverage because they represented missing regions of fragmented loci. Phylogenetic comparisons among proteins relied on ClustalOmega alignments [33] and Neighbor joining trees generated with the web-based interface at EBI [34] available at http://www.ebi.ac.uk or using the MUSCLE + PhyML pipeline available at http://www.phylogeny.fr.

The gapped contigs were filtered to remove contaminating sequences, and sequences less than 200 nt prior to depositing into public databases. Sequence Reads, Gapped Contigs, and Predicted Proteins are all stored in public databases under BioProject PRJNA350546.

## Results and discussion

### Genome size and assembly metrics

The *E*. *maynei* genome size has not been reported. Kmer distributions from our survey data provided the first estimates of this metric. The genome size estimates, based on the two Kmer genie models, ranged from ~68 to ~97 Mb (S1 Fig). Thus, the survey provided >30x coverage of the sequenceable genome.

Inbred or isogenic lines of *E*. *maynei* have never been generated. The *E*. *maynei* genome survey was based on a pool of DNA from thousands of (presumably genetically different) individual mites. Current assembly methods have trouble with heterogenous DNA samples such as this. However, stretches of unique sequences (like protein-coding genes) are more amenable to reconstruction than repetitive elements. As a result, we anticipated that genes that were not interrupted by repetitive elements or that did not possess gross allelic variation within the population (large insertions or deletions) would be able to be assembled from the sequence data.

Assembly metrics for scaffolds are presented in S1 Table. BUSCO was performed on assembled contigs to test how well genes were assembled from the data, and to estimate how well the assemblies represented a complete gene set. These analyses indicated that the Minia-produced assemblies using a kmer length of 47 or 53 were the best in terms of the number of complete or fragmented loci present, and with the fewest missing loci (Table 1). The models used by Kmer genie predicted that these assemblies would be the best. SSPACE Scaffolds (gapped contigs) generated with these two data sets displayed improvement over the contigs, and were also better than the scaffolds generated by CLC assembly methods. The scaffolds from the Minia/SSPACE assembly using a kmer length of 47 were then selected for use in protein predictions. The size of this assembly was roughly 59 Mb, distributed across 196,133 fragments with an N50 of 480 nt. Following removal of short (<200nt) sequences, the deposited assembly represented 43 Mb across 72,749 fragments with an N50 of 788.

**Table 1:** BUSCO analyses of contigs, scaffolds, and predicted proteins for *Euroglyphus maynei*.

Test	k	m	Complete	Duplicated	Fragmented	Missing	# loci	Lineage / mode
**Contigs**								
	21	17	3.7	0.2	9.3	86	429	Eukaryota / genome
	47	7	26	1.1	17	56	429	Eukaryota / genome
	53	12	23	1.1	12	64	429	Eukaryota / genome
	71	7	15	0	12	72	429	Eukaryota / genome
	91	3	3.2	0	8.8	87	429	Eukaryota / genome
**Scaffolds**								
	47	7	31	1.3	17	50	429	Eukaryota / genome
	53	12	26	1.3	14	58	429	Eukaryota / genome
	25 (CLC Bio)	n.a.	32	5.3	10	56	429	Eukaryota / genome
**Proteome**								
	47	7	61	11	29	9	429	Eukaryota / OGS
	47	7	43	3.6	25	30	843	Metazoa / OGS
	47	7	32	2.4	18	49	2675	Arthropoda / OGS

Values for kmer length (k), minimum abundance (m) during assembly, and the percentages of complete, duplicated, fragmented and missing loci are presented along with the total number of loci sampled, the analysis lineage and mode used.

### Predicted proteome

Maker predicted over 15,000 proteins (or fragments). The predicted proteome was also analyzed by BUSCO and nearly double the number of complete loci were identified when compared to the tests of scaffolds. This likely represented enhancements that the Maker pipeline provided for gene predictions and structural annotations which were not inherent in the methods embedded in the BUSCO genomic DNA analysis pipeline. BUSCO estimates of missing proteins ranged from 9% of highly conserved eukaryotic loci to 49% of arthropod (mostly insect) loci. However, prior analyses suggest that the number of missing loci is overestimated when BUSCO is performed with species of acari [7,35]. Genome statistics generated by GAG revealed that the majority of predicted proteins (80.4%) had a start codon, but that only 8,198 of the 15,598 loci (52.5%) had both a predicted start codon and a predicted stop codon. All analyses indicated that the assembled *E*. *maynei* sequences were partly fragmented, and that some loci were probably missed in our survey (Table 1). Although the assembly and gene prediction methods had some difficulty assembling and predicting full-length genes from the source sequences, the majority of test loci were represented in our data set.

### Comparison to related mites

**Fig 1:**
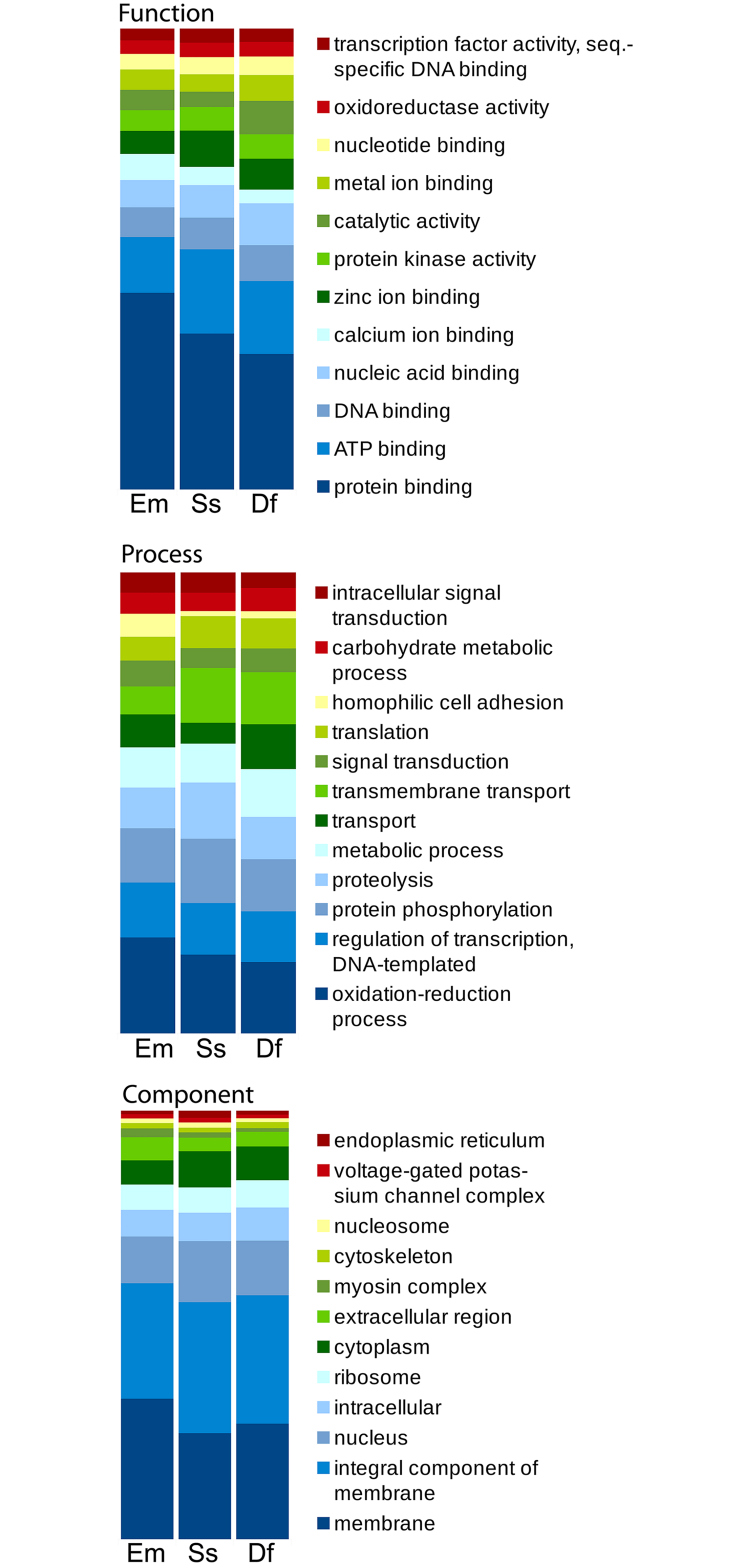
Top Gene Ontologies for mite proteins containing Interpro domains. The twelve most abundant GO terms were identified for *E*. *maynei* (Em) and the corresponding values for those terms for *S*. *scabiei* (Ss) and *D*. *farinae* (Df) were compiled. Data for Cellular Processes, Cellular Components, and Molecular Functions are presented. Bar volumes represent the percentage of proteins annotated with a particular GO term when compared to the total number of proteins in the twelve selected categories for each species.

**Fig 2:**
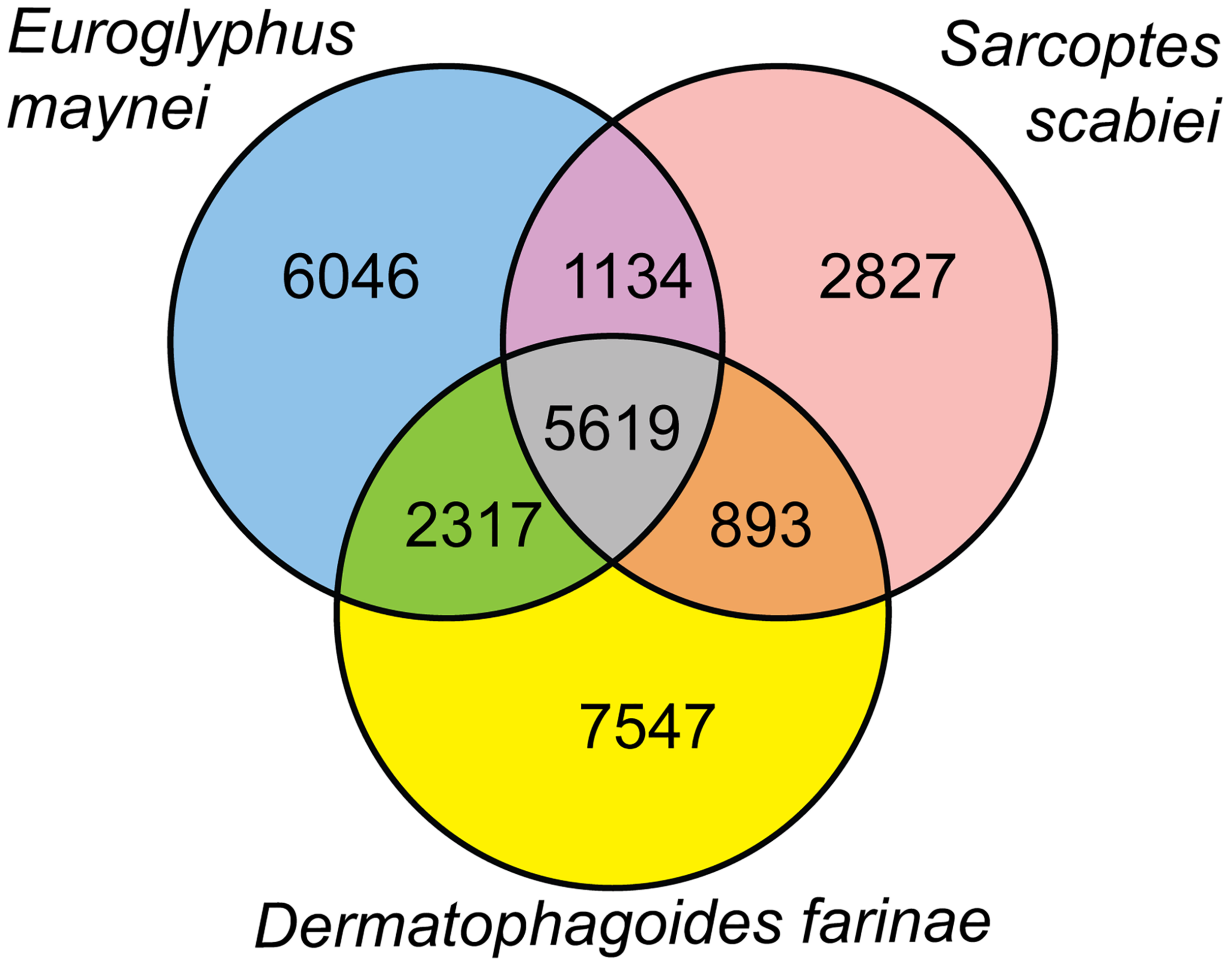
Venn diagram representing shared orthologs in three astigmatid mite species. Orthologs were identified as best reciprocal Blast hits.

To determine if the assembled genome and predicted proteome were representative of an astigmatid mite, we made multiple comparisons. The assembled genome of 59 Mb was similar to the genomes assembled from *S*. *scabiei* (56.2 Mb) and *D*. *farinae* (53.5 Mb), two other mites in the Astigmata. Previous analyses indicated that astigmatid mite genomes are replete with simple sequence repeats [7]. We clearly demonstrated that simple sequence repeats (SSR) are more frequent in the genomes of astigmatid mites than they are in the genomes of ticks and other non-astigmatid mites whose genomes are larger. This is not the same phenomenon as having a large proportion of the total genome represented by transposons and retroviral sequences. The *E*. *maynei* genome was found to have >1400 SSR/megabasepair (S2 Table). This number was slightly over half of that found in either *S*. *scabiei*, or *D*. *farinae*, but it was consistent with what we expected from an astigmatid mite [7]. Blast2Go was used to categorize predicted proteins into molecular functions, biological processes, and cellular components for comparison to the *S*. *scabiei* and *D*. *farinae* proteomes. The most abundant molecular functions, biological processes, and cellular components were not substantially different across the mite species and were consistent with their phylogenetic relationship (Fig 1). We also utilized reciprocal best BLAST searches of *D*. *farinae*, *S*. *scabiei*, and *E*. *maynei* to identify proteins that were common across these astigmatid mite species. There were more than five thousand proteins shared among the predicted proteomes of the three astigmatid mites that were examined, but over 25% of the loci from each species were not able to be assigned to an ortholog in another species using reciprocal BLAST (Fig 2). The unassigned loci included homologs, as well as unique sequences.

### Allergen homologs

Information from *E*. *maynei* can aid our understanding of the evolution and molecular biology of other mites such as *Dermatophagoides farinae* and *Sarcoptes scabiei*. However, a key impetus for studying *E*. *maynei* is that it is an important source of allergens for humans and companion animals. In the future, diagnostic tests and immunotherapy will likely be based on using recombinant allergens. Thus, knowledge of the *E*. *maynei* genome, and allergens predicted to be encoded by it are prerequisites for developing these. Because limited molecular information is available for *E*. *maynei* allergens, we were interested in identifying *E*. *maynei* orthologs of the previously reported dust mite allergens.

Mite allergens are placed into groups based on their immunoreactivity and the groups are named sequentially based on date of discovery. There are currently 33 allergen classes (Group 1 –Group 33) associated with house dust mites [36]. The WHO/IUIS database (http://www.allergen.org/) attempts to maintain an authoritative listing of these allergens, but ambiguities persist in the nomenclature for the more recently described mite allergens. This stems from discord among published manuscripts, the presence of sequence database entries from different investigators, a lack of sequence data, or a combination of these issues. For example, Group 17 is reported to be a Calcium-binding EF hand protein [37], but no sequence data has been deposited in a publicly available database for comparisons. Three separate identities have been given to Group 26: EF-2 [38], cyclophilin [39], or myosin [36]. The latter is presented in a review that cites An et al. [38]. However, a more recent review by the same author [40] cites the WHO/IUIS database. Two different assignments have been made for Group 29: cyclophilin [38] or Profilin (Accession number AIO08866.1) [39]. Furthermore, the WHO/IUIS database indicates that myosin and ferritin were previously alternatives for the group 30 allergens. From a bioinformatics perspective, it was straight forward to identify even distantly related sequences when sequence data was available, but assigning sequences to the proper allergen group name (even when clearly orthologous proteins were identified) was problematic. We were able to identify sequences encoding allergen candidates for 28 of the 30 allergen groups for which sequence data have been unequivocally assigned, as well as candidates for alternative group 26 and group 29 proteins (EF-2, cyclophilin, profilin). Of the 30 allergen groups, we were only unable to find group 19 (a short antimicrobial peptide) and group 24 (ubiquinol-cytochrome c reductase binding protein) in our data set.

Two groups did not assemble well as evidenced by incomplete or fragmented protein coding regions: gelsolins (Group 16) and the Heat Shock Proteins (Group 28). Gelsolin proteins contain a repeat structure, and this likely created ambiguity during assembly of sequences into full-length protein coding loci. With the Heat Shock Proteins, a similar assembly challenge existed but it was likely due to the presence of a multigene family. This also occurred for certain gene families in the *S*. *scabiei* genome, where the same assembly strategy was used [7].

Additional allergen candidates were split among multiple contigs. For example, a single paramyosin was represented by three contigs, as was one of at least two identified apolipophorin genes. An amylase gene was split in two, as was an inorganic pyrophosphatase. A supervised assembly method would likely result in a more complete reconstruction of these loci. The majority of loci with homology to known allergens were assembled as contiguous sequences and contained complete functional domains (Table 2).

**Table 2:** Allergen homologs in the genome of *E*. *maynei*.

Locus ID	Start Methionine	Complete Domains	Fragmented Domains	Orthology
BLA29_000749-RA	Yes	I29, Peptidase C1		Yes
BLA29_005376-RA	Yes	I29, Peptidase C1		Yes
BLA29_005717-RA	Yes	I29, Peptidase C1		Yes
BLA29_002725-RA	Yes	I29, Peptidase C1		
BLA29_000188-RA	Yes	Peptidase C1		
BLA29_005995-RA	No	Peptidase C1		
BLA29_001679-RA	No	Peptidase C1		
BLA29_002474-RA	Yes	Peptidase C1		
BLA29_004167-RA	Yes	Peptidase C1		
BLA29_009596-RA	No	Peptidase C1		
BLA29_007497-RA	Yes	Peptidase C1		
BLA29_001844-RA	Yes	ML superfamily		Yes
BLA29_002039-RA	Yes	ML superfamily		Yes
BLA29_007771-RA	Yes		ML superfamily	
BLA29_004181-RA	Yes	ML superfamily		
BLA29_001646-RA	Yes	ML superfamily		Yes
BLA29_001231-RA	Yes	Tryp SPc		Yes
BLA29_009032-RA	Yes	Tryp SPc		
BLA29_000748-RA	Yes	Tryp SPc		
BLA29_004152-RA	Yes	Tryp SPc		
BLA29_004244-RA	Yes	Tryp SPc		
BLA29_003534-RA	Yes	Tryp SPc		
BLA29_001429-RA	Yes	Tryp SPc		
BLA29_001029-RA	Yes	Tryp SPc		
BLA29_004415-RA	Yes	Tryp SPc		
BLA29_005972-RA	Yes	Tryp SPc		
BLA29_003326-RA	Yes	Tryp SPc		
BLA29_006839-RA	No	Tryp SPc		
BLA29_007935-RA	Yes	Tryp SPc		
BLA29_008722-RA	No	Tryp SPc		
BLA29_008667-RA	No	Tryp SPc		
BLA29_004732-RA	Yes	TSP 1, Tryp SPc		
BLA29_013905-RA	No		Amy Ac	
BLA29_003192-RA	Yes	Amy Ac, Amy C		
BLA29_005982-RA	No		Amy Ac, Amy C	
BLA29_010118-RA	Yes	Blo t 5		Yes
BLA29_005095-RA	Yes	Tryp SPc		Yes
BLA29_008589-RA	Yes	Grp7 allergen		
BLA29_008391-RA	Yes	Grp7 allergen		
BLA29_010568-RA	Yes	GST N Mu, GST C		
BLA29_007805-RA	Yes	GST N Mu, GST C		
BLA29_008641-RA	Yes	GST N Mu, GST C		
BLA29_002590-RA	Yes	Tryp SPc		Yes
BLA29_005861-RA	Yes		Tropomyosin	Yes
BLA29_002808-RA	Yes		Myosin tail 1	Yes 1/3
BLA29_004720-RA	No		Fam76, TMPIT, Bap31	Yes 1/3
BLA29_010257-RA	No		Myosin tail 1	Yes 1/3
BLA29_002507-RA	Yes	Glyco 18, CBM 14		
BLA29_002269-RA	No	Glyco 18, CBM 14, CBM 14		
BLA29_005952-RA	Yes	Glyco 18		
BLA29_014546-RA	Yes	CBM 14		
BLA29_003622-RA	Yes	CBM 14		
BLA29_011891-RA	No	CBM 14, CBM 14		
BLA29_012640-RA	Yes	Lipocalin		Yes
BLA29_007304-RA	Yes	Lipocalin		
BLA29_007408-RA	Yes		Lipocalin	Yes
BLA29_007657-RA	Yes	Lipocalin		Yes
BLA29_008468-RA	Yes		Lipocalin	Yes
BLA29_010085-RA	No	Lipocalin		Yes
BLA29_010726-RA	Yes			Yes 1/3
BLA29_003031-RA	Yes	LPD N		Yes 1/3
BLA29_002649-RA	Yes			Yes 1/3
BLA29_008619-RA	Yes	ADF Gelsolin	ADF Gelsolin	
BLA29_014251-RA	No		ADF Gelsolin, ADF Gelsolin	
BLA29_001338-RA	Yes	arginine kinase like		Yes
BLA29_006943-RA	No	arginine kinase like		Yes 1/2
BLA29_011280-RA	Yes		arginine kinase like	Yes 1/2
BLA29_006646-RA	Yes	arginine kinase like		
BLA29_006289-RA	Yes	Blo t 5		Yes
BLA29_010079-RA	Yes		TIM phosphate binding	
BLA29_008286-RA	No	Serpin		
BLA29_005081-RA	Yes	Serpin		
BLA29_002584-RA	Yes		Serpin	
BLA29_005776-RA	Yes	Serpin		
BLA29_000753-RA	Yes	Serpin		
BLA29_004510-RA	Yes	Serpin		
BLA29_004546-RA	Yes		Serpin	
BLA29_002279-RA	Yes		Serpin	
BLA29_011336-RA	Yes		HSP70	
BLA29_015195-RA	Yes		HSP70	
BLA29_006000-RA	No		HSP70/dnaK	
BLA29_008310-RA	Yes		HSP70	
BLA29_008418-RA	No		HSP70	
BLA29_003383-RA	Yes	HSP70		
BLA29_006210-RA	Yes		HSP70	
BLA29_001944-RA	Yes		HSP70	
BLA29_004075-RA	Yes		HSP70	
BLA29_004373-RA	Yes		HSP70	
BLA29_008613-RA	Yes		HSP70	
BLA29_002587-RA	Yes	Cyclophilin, RRM PPIL 4		
BLA29_010596-RA	Yes	Cyclophilin		
BLA29_003954-RA	No	Cyclophilin		
BLA29_009322-RA	Yes	Cyclophilin		
BLA29_010263-RA	Yes	Cyclophilin		
BLA29_012512-RA	No		Cyclophilin	
BLA29_013856-RA	Yes		Cyclophilin	
BLA29_006024-RA	Yes	Cyclophilin		
BLA29_007484-RA	Yes	Cyclophilin		
BLA29_008744-RA	No	Cyclophilin		
BLA29_002533-RA	Yes	WD40, Cyclophilin		
BLA29_004461-RA	Yes	RRM PPIE, Cyclophilin		
BLA29_000133-RA	Yes	PROF		
BLA29_008125-RA	Yes	Ferritin		
BLA29_002580-RA	Yes	Ferritin		
BLA29_008226-RA	Yes		Ferritin	
BLA29_001133-RA	Yes	ADF Cofilin		Yes
BLA29_004452-RA	No		Pyrophosphatase	
BLA29_014471-RA	Yes		Pyrophosphatase	
BLA29_003724-RA	Yes	alpha tubulin		
BLA29_003856-RA	Yes	alpha tubulin		
BLA29_001366-RA	Yes	alpha tubulin		

The locus ID for each allergen identified is indicated, along with whether or not it had a predicted start codon. Complete and partial functional domains are indicated. Orthology was determined by phylogenetic analyses of gene families.

### Allergen orthologs

There are different allergen groups that either share sequence homology between groups, or that are represented by multigene families. Therefore, an additional challenge is to identify the best allergen candidates from among those that show some homology to the known allergens. To do this, we pursued a phylogenetics approach within certain gene families to identify which *E*. *maynei* homologs were most closely related to the known allergens (Table 2). Noteworthy results from those comparisons are presented below.

#### Cysteine proteases (group 1 family)

The cysteine proteases in *E*. *maynei* were represented by at least 11 proteins with homology to cathepsins and allergenic proteases from other species. All possessed a complete peptidase domain. One of these cysteine proteases was orthologous to the two known allergens from *Dermatophagoides* mites (Der f 1 and Der p 1). Two other proteins also displayed close affinity to these allergens in the phylogenetic analyses. The other 9 homologs were more distantly related and were outside the clade that includes the expanded family of scabies allergen paralogs [7]. In *S*. *scabiei*, the majority of cysteine protease allergen paralogs that have been identified have mutations that inactivate the protein and it has been proposed that these scabies mite inactive protease paralogs (SMIPP-C) are involved in modulating or evading the host immune system [41]. Only one protein in *E*. *maynei* was missing the active site cysteine and one of the predicted proteins lacked a start methionine. These observations were consistent with the hypothesis that inactive protease genes are under a unique type of selection pressure in scabies mites that is not present in house dust mites like *E*. *maynei* and *D*. *farinae*.

#### Lipid binding domain proteins (group 2 and 22 families)

MD-2 like lipid binding domains are present in two different allergen groups (2 and 22). Group 2 is considered to be a major allergen, and group 22 is considered a minor allergen. In *E*. *maynei*, the predicted proteome contained four proteins with complete MD-2 domains and one protein with a partial domain. Two of these proteins were clear orthologs of Der f 2, and one was closely related to Der p 22. The remaining two homologs (one was the partial protein) were more similar to family members from *Sarcoptes scabiei*.

#### Serine proteases (group 3, 6, and 9 families)

Twenty serine protease homologs were identified. In *Sarcoptes scabiei*, the serine protease gene family is expanded and contains inactive paralogs that are speculated to be involved in manipulating host immunity [42–44]. Most of the serine protease allergen homologs found in *S*. *scabiei* var. *canis* are derived from group 3 allergens and are present as tandem duplications in the genome [7]. In *E*. *maynei*, there was one protein that appeared to be an ortholog to each of the group 3, 6, and 9 allergens from *Dermatophagoides* mites. Each of these three proteins possessed an intact (active) catalytic triad. There were 17 more predicted proteins whose relationships were unclear. They were from different genomic scaffolds and they were attached by long branches to the main phylogenetic tree (S2 Fig). Other than being related to the allergens, no attempt has been made to determine what type of proteases these are. Two of the predicted proteins contained short introns and may represent pseudogenes. Three of the remaining proteins were missing one or two of the residues in the catalytic triad, and are likely inactive. A different subset of three proteases appeared to be missing the residues required for substrate binding. Thus, the serine protease gene family in *E*. *maynei* was unlike that of either *Dermatophagiodes* or scabies mites.

#### Amylases (group 4 family)

Mite group 4 allergens are amylase enzymes that can be found in feces. A nearly full length *E*. *maynei* homolog was identified that is a little different from the existing NCBI record (AAD38943; 82% identity). Two other fragments were found that probably represent the N and C termini of the existing NCBI record (>98% identity). So, at least two alpha amylases were present in *E*. *maynei*, one of which is the previously identified ortholog to the group 4 allergens. This is in contrast to *S*. *scabiei*, which did not appear to possess an ortholog of the allergenic alpha amylase, but did possess distantly related amylases [7].

#### Chitin-interacting proteins (group 12, 15, 18, 23 families)

Four groups of allergens, also present in mite feces, share the presence of chitin interaction domains. One domain is a chitin-binding module (CBM14; present in all four allergen groups) and the other is a putative chitinase domain (Glyco18; also found in groups 15 and 18). Three of the proteins in *E*. *maynei* that showed homology to these allergens contained only the CBM14 domain, one contained only a Glyco18 domain, and two possessed both Glyco18 and CBM14 domains. The domain organization, and the homology of the *E*. *maynei* proteins to known allergens is discordant. While these might be potential *E*. *maynei* allergen candidates, none of these proteins could be assigned as orthologs to the known mite allergens.

#### Fatty acid binding proteins (group 13 family)

There were 6 homologs of the group 13 allergens. One was most closely related to a version of Blo t 13, and one was most closely related to Der p 13. Three of the remaining 4 appeared to be paralogs, and along with one *S*. *scabiei* protein formed a clade with Der f 13 (S3 Fig).

#### Arginine kinases (group 20 family)

Four arginine kinase-containing contigs were identified that probably represent three loci. Two proteins appear to be paralogs (77% identity) with one of the paralogs split into two contigs that were probably fragmented during assembly because of repetitive sequences (as was the case in *S*. *scabiei*). These paralogs appeared to be orthologs of the *Dermatophagoides* allergens, while the last arginine kinase was a more distantly related type.

#### Serpin-like proteins (group 27 family)

Eight serpin like proteins were identified. Only one could be considered a candidate ortholog for Der f 27, and the remainder were more distantly related.

#### Alpha tubulin homologs (group 33 family)

Three alpha tubulin homologs were identified. All were very closely related to one another and to other alpha tubulins. The high sequence conservations made them indistinguishable from the perspective of determining which one is a more likely allergen candidate.

## Conclusions

The *E*. *maynei* draft genome is similar to other astigmatid mites because it is small and possesses a large number of simple sequence repeats per megabase pair. Future investigations will undoubtedly improve the assembly and annotation, which could be achieved with alternative DNA sources (e.g., sequences derived from multiple individual mites, or using long read technologies). Gene annotations would also be improved with the acquisition of RNA sequence data. We found that the composition of the predicted proteomes from astigmatid mites were similar with respect to the proteins that were able to be categorized based on gene ontology term abundance for conserved domains. Allergen-related proteins were readily identified within the predicted proteome. Identifying clear 1:1 orthologs for the mite allergen proteins was more challenging. The sequence data generated from this study should allow for the development of better methods for diagnosing the sources of allergens originating from different species of astigmatid mites that occur in house dust in homes worldwide. It is also a prerequisite for developing well-defined recombinant allergen cocktails for the treatment of dust mite allergies. The work is timely and important to enable new approaches for the diagnosis and understanding of the mechanistic aspect of the allergic reaction to house dust mites.

## Supporting information

S1 Fig Kmer analyses of trimmed reads from *E*. *maynei*. The top right is the data output from the standard model test with the optimal kmer distribution graph presented to the right. Bottom is the data output from the diploid model test with the optimal kmer distribution to the right. In both models, the model is represented by the red line. For the diploid model, the green line represents the heterozygous kmer distribution and the blue line represents homozygous kmer distribution predicted within the model.
(TIF)

S2 Fig Maximum likelihood tree for the group 3, 6 and 9 mite allergens (serine proteases). Bootstrap values are included.
(TIF)

S3 Fig Maximum likelihood tree for the group 13 mite allergens and related proteins (fatty acid binding proteins). Bootstrap values are included.
(TIF)

S1 Table Assembly metrics for contigs before scaffolding. (XLSX)

S2 Table MISA output data file for simple sequence repeat counts. (XLSX)
