# Supplementary figures and images for: Allergen homologs in the Euroglyphus maynei draft genome
Source: PLoS One. 2017 Aug 22;12(8):e0183535. doi: 10.1371/journal.pone.0183535 (PMC5568732; doi:10.1371/journal.pone.0183535)

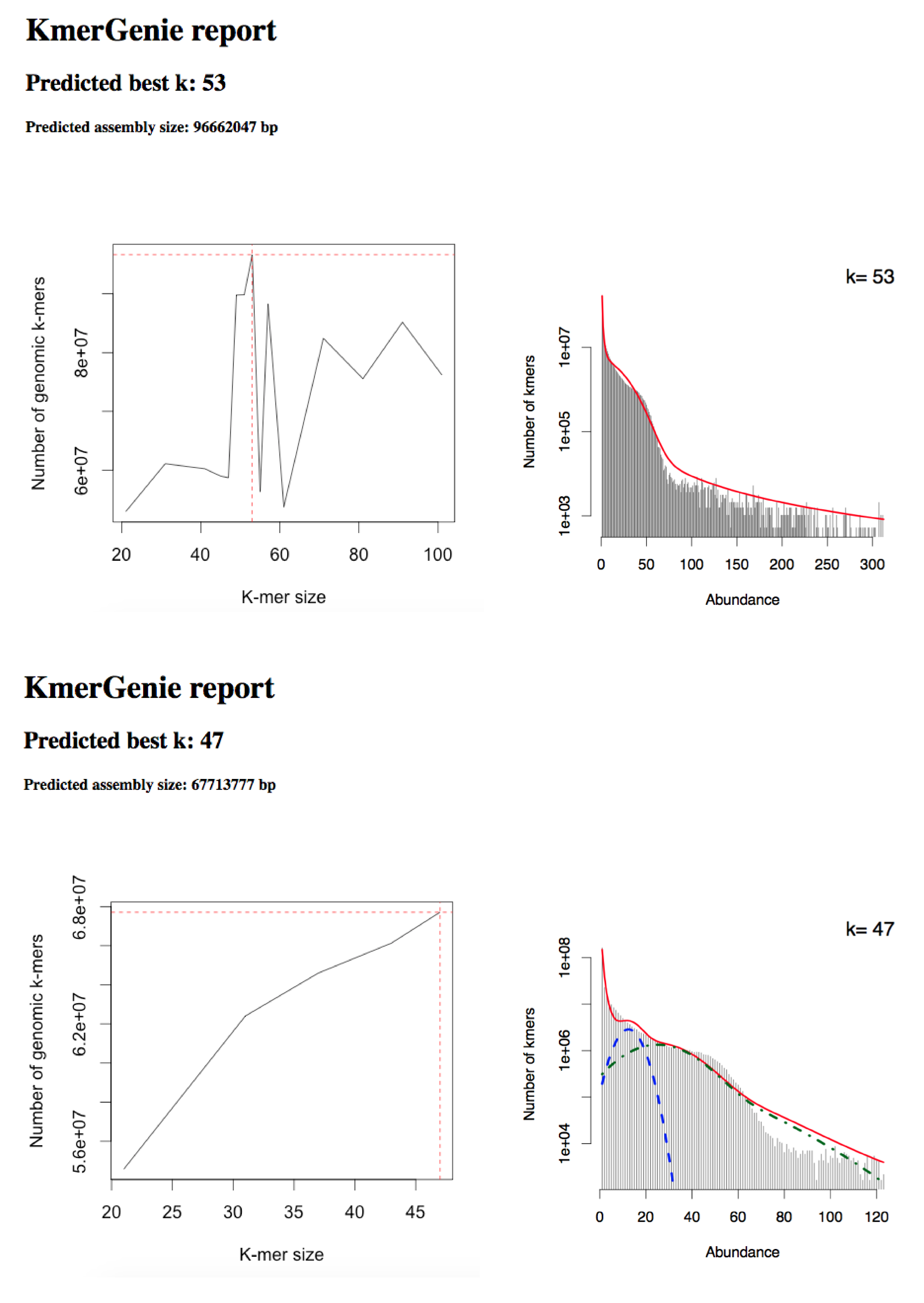

Supplement: S1 Fig — The top right is the data output from the standard model test with the optimal kmer distribution graph presented to the right. Bottom is the data output from the diploid model test with the optimal kmer distribution to the right. In both models, the model is represented by the red line. For the diploid model, the green line represents the heterozygous kmer distribution and the blue line represents homozygous kmer distribution predicted within the model. (TIF) [file pone.0183535.s001.tif]

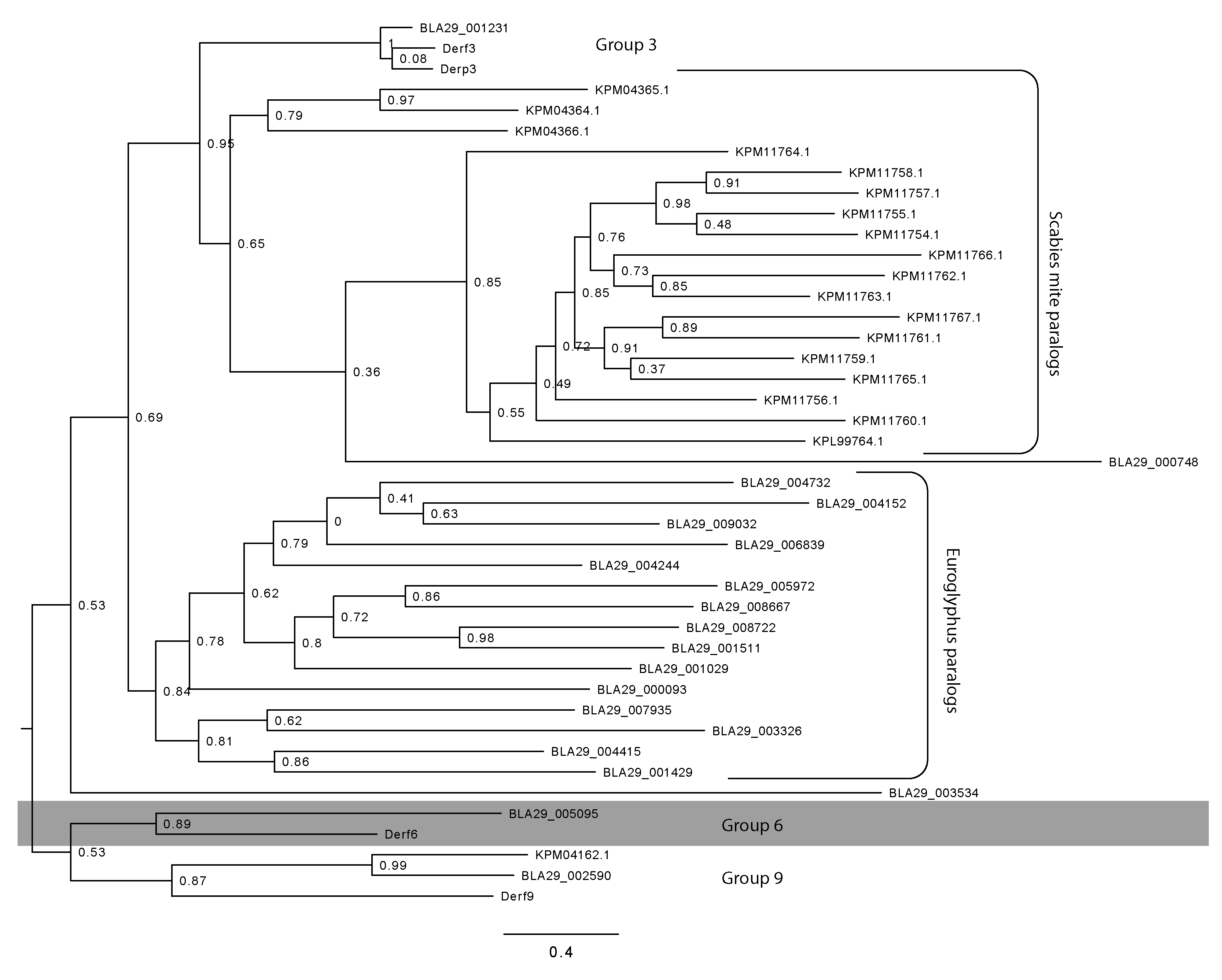

Supplement: S2 Fig — Bootstrap values are included. (TIF) [file pone.0183535.s002.tif]

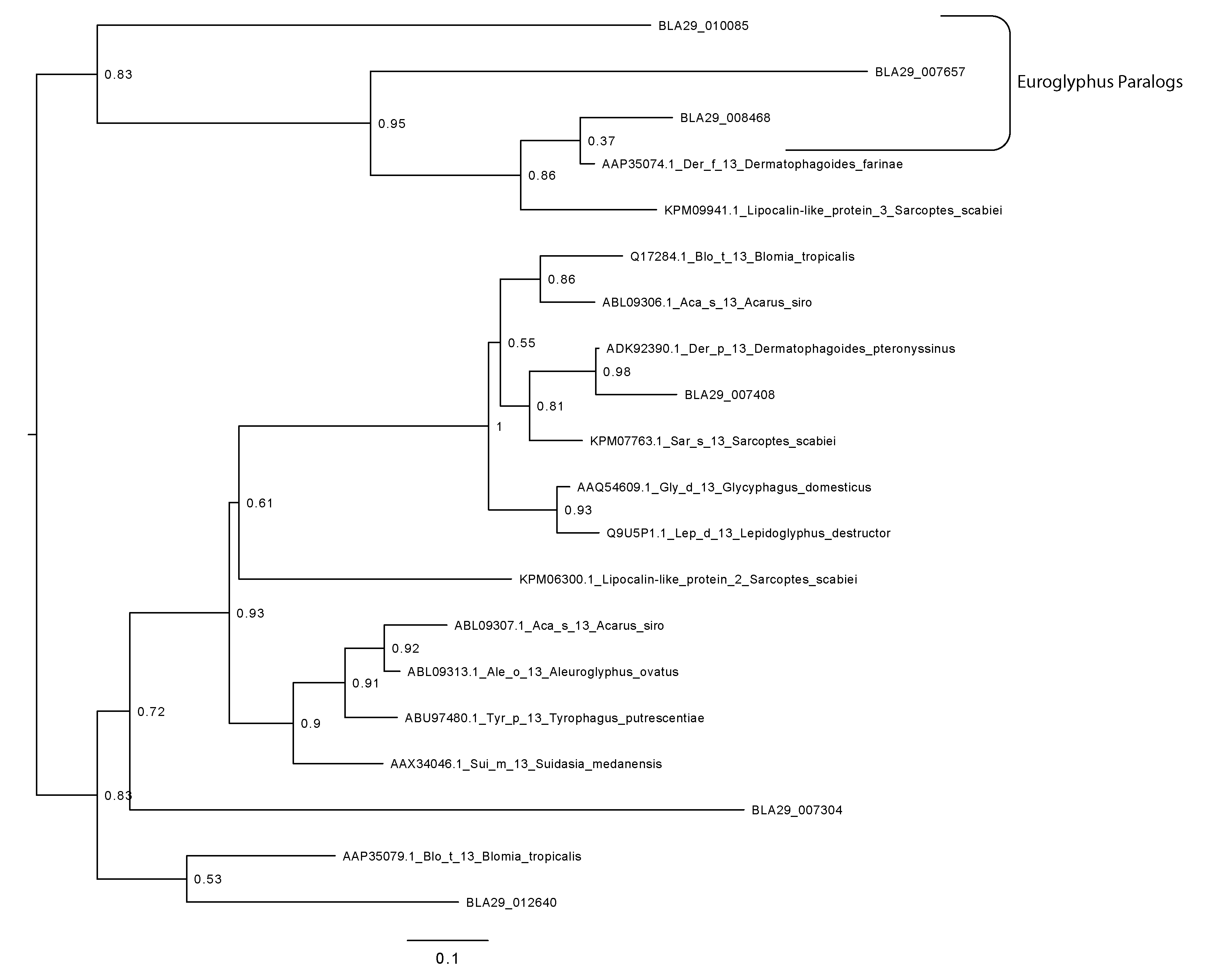

Supplement: S3 Fig — Bootstrap values are included. (TIF) [file pone.0183535.s003.tif]
